# Deciphering the Molecular Mechanism of Red Raspberry in Apoptosis of Liver Cancer Cells

**DOI:** 10.1155/2022/2026865

**Published:** 2022-04-27

**Authors:** Linlin Song, Qi Li, Hui Shi, Hui Yue

**Affiliations:** ^1^College of Pharmacy, Jiamusi University, Jiamusi 154007, Heilongjiang, China; ^2^Department of Biochemistry, Mudanjiang Medical University, Mudanjiang 157011, Heilongjiang, China; ^3^Pharmacy Department, First Affiliated Hospital of Jiamusi University, Jiamusi 154007, Heilongjiang, China; ^4^Department of Pathology, Mudanjiang Medical University, Mudanjiang 157011, Heilongjiang, China

## Abstract

Red raspberry contains a variety of bioactive ingredients and has high edible and medicinal value. Red raspberry extractions (RREs) have strong antioxidant capacity and anticancer ability in vivo and in vitro. This study was to explore the specific mechanism of RREs inhibiting the proliferation of liver cancer HepG2 cells and provide a theoretical basis for the prevention and treatment of liver cancer by RREs. HepG2 cells were cultured in vitro, and MTT assay was adopted to detect the effect of RREs on HepG2 cell activity. Colony formation assay was applied to detect the growth and proliferation of cells, cell apoptosis was detected by flow cytometry, and dichloro-dihydro-fluorescein diacetate (DCFH-DA) assay was adopted to detect the effect of RREs on the production of reactive oxygen species (ROS) in cells. The effect of RREs on cell mitochondrial membrane potential was evaluated by mitochondrial membrane potential assay kit with JC-1 (JC-1 assay), and western blot was used to detect the expression of apoptosis-related proteins (B-cell lymphoma-2 (Bcl-2), Bcl-2-associated *x* (Bax), and Caspase-3), thus investigating the effect of RREs on the molecular mechanism of HepG2 cell apoptosis. The results showed that RREs could inhibit the proliferation activity of HepG2 cells and promote their apoptosis in a concentration-dependent manner. The level of ROS in HepG2 cells interfered by RREs increased markedly, while the cell mitochondrial membrane potential decreased sharply. As the concentration of HepG2 increased, the mitochondrial membrane potential reduced steeply. Western blot results showed that the expression of apoptosis-related protein Bcl-2 in the RREs treatment group dropped, but the expression of Bax and Caspase-3 rose. In summary, RREs could inhibit the proliferation of liver cancer HepG2 cells and promote their apoptosis. This inhibition might be executed by inducing HepG2 cells to produce ROS, a decrease in Bcl-2/Bax protein ratio, and an obvious reduction in mitochondrial membrane potential.

## 1. Introduction

Cancer is a serious threat to human life and health. However, radiotherapy and chemotherapy, the main methods of cancer treatment, have certain side effects and limitations [1]. Therefore, it is imperative to develop new drugs and treatment methods. Studies suggested that there are two different manifestations between tumor cells and normal cells. On the one hand, tumor cells are under oxygen pressure due to relatively high ROS content [2, 3]. On the other hand, tumor cells contain lower enzymes and nonenzymatic antioxidants. Thus, the two different manifestations make tumor cells more sensitive to ROS [4, 5]. Besides, the existence of these two differences provides a biochemical basis for selectively killing tumor cells by increasing ROS [6]. The free radicals are used for selective antitumor to achieve therapeutic effects [7, 8], which have attracted more and more attention from researchers.

Red raspberry contains a lot of active substances, such as phenolic acids, flavonoids, proanthocyanidins, and ellagic acid [9, 10]. Proanthocyanidins and ellagic acid have certain preventive and therapeutic effects on tumors [11]. At present, most of the domestic research on red raspberries focus on the identification of active ingredients and the separation of biologically active substances [12, 13], and there are relatively few studies on the specific anticancer mechanisms. It is expected that RREs can prevent and treat oxidative stress-related diseases by protecting the body through antioxidant effect. In addition, ROS can be used to selectively kill cancer cells to treat cancer for antioxidants can promote oxidation under certain conditions. To gain a deeper understanding of antioxidant and oxidative stress, and to better understand the relationship between free radicals and tumors, the specific mechanism of RREs inhibiting the proliferation of liver cancer cells was explored in this work, hoping to provide new ideas for the prevention and treatment of liver cancer.

## 2. Materials and Methods

### 2.1. Cell Culture

Human liver cancer cell line HepG2 was purchased from Shanghai Honsun Biological Technology Co., Ltd., China. HepG2 cells were cultured in a 37°C and 5% CO_2_ incubator with the Roswell Park Memorial Institute (RPMI) 1640 complete medium (containing 10% fetal bovine serum, 100 *μ*g/mL penicillin, and 100 *μ*g/mL streptomycin). Besides, the medium was changed every two days. When the confluence of the cells reached 70%–80%, trypsin was used for digestion and passage or experiments.

### 2.2. Extraction Method of Red Raspberry

The frozen red raspberry fruit was taken out, thawed at room temperature (25°C), and pulped. There was reflux and extraction for three times (material-to-liquid ratio was m(g): V(mL) = 1 : 8, temperature was 60°C, and extraction solvents were distilled water, 50% ethanol solution, and 100% ethanol solution in turn). Then, it was centrifuged at 3,000 r/min for 10 minutes, combined with the supernatant, and distilled under reduced pressure. Besides, the concentrates were combined, and the mixture was frozen and dried. Finally, the collected RREs were stored at −80°C.

### 2.3. MTT Assay

The effect of RREs on the cell activity of human liver cancer cell line HepG2 was analyzed. Cells were inoculated in a 96-well plate at 2 × 10^4^ cells/well, and 200 *μ*L RPMI 1640 culture medium was added to each well. After the cells adhered to the wall, the RREs were added at the concentration of 0, 5, 10, 20, 60, 80, 120, and 160 mg/mL. Six auxiliary wells were set up in each group. 48 hours after the addition of the drug, 20 *μ*L of MTT was added to each well under dark conditions. After four hours of incubation, the medium and MTT were discarded. Then, 150 *μ*L dimethyl sulfoxide (DMSO) was added to each well, which was shaken for 5 minutes, and the optical density (OD) of each well was measured at the absorbance of 490 nm. The inhibition rate of different concentrations of drugs on cell activity was calculated, and the half inhibitory concentration of RREs on the activity of HepG2 cells was also calculated. Besides, the growth inhibition of the cells was measured 48 hours after the drug was added, and the cell inhibition rate of different concentrations of RREs was calculated. Cell inhibition

(1) $$\begin{array}{c} \text{rate}=\frac{\text{experimental group}}{\text{blank control group}}\times 100\text{\%.} \end{array}$$

### 2.4. Clone Formation Experiment

Cloning experiments were performed using the method in reference [14]. The effect of RREs on the growth and proliferation status of cells were observed. The cells were inoculated in a 6-well plate at 1,000 cells/well, and 3 mL culture medium was added to each well. After the cells adhered to the wall, a blank group, a 30 mg/mL RREs group, and a 60 mg/mL RREs group were set up. Next, the RREs groups were added with different concentrations of drugs, and the blank group was added with an equal volume of normal saline. The culture solution was discarded after 14 days of culture, and phosphate buffer saline (PBS) was utilized to rinse the cells carefully for twice. Each well was added with 5 mL of 4% paraformaldehyde to fix the cells for 15 minutes. Then, the fixative was discarded, and the above was rinsed with distilled water twice and added with an appropriate amount of crystal violet staining solution to soak for 15 minutes. The staining solution was slowly washed off with running water, and then it was dried in the air. After picture taking, the software was adopted to count the number of colonies formed by the clones. Clone formation

(2) $$\begin{array}{c} \text{rate}=\frac{\text{number of clones forming cell colony}}{\text{number of inoculated cells}}\times 100\text{\%.} \end{array}$$

### 2.5. Cell Apoptosis Detected by Flow Cytometry

The method in reference [15] was used to detect apoptosis of HepG2 cells in each group. HepG2 cells grown in the logarithmic phase were inoculated in a 6-well plate at a concentration of 3 × 10^5^ cells/mL and cultured in an incubator with 2 mL per well at 37°C and 5% CO_2_ saturated humidity for 24 hours. Then, the control group, 30 mg/mL RREs group, and 60 mg/mL RREs group were set up. After a culture of 48 hours, the cells were collected, washed with precooled PBS 3 times, and added with 195 *μ*L Annexin V-FITC binding solution. The cells were gently resuspended, and 5 *μ*L Annexin V-FITC and 10 *μ*L of propidium iodide staining solution were added, which was mixed gently. Finally, cells were incubated at room temperature under dark conditions for 10–20 minutes and then detected by flow cytometry.

### 2.6. Cell ROS Detection

ROS levels in HepG2 cells in each group were detected using the method in reference [16]. The HepG2 cells growing in the logarithmic phase were collected for resuspension and inoculated in a 6-well plate at a density of 3 × 10^5^ cells/mL. An untreated group, a 30 mg/mL RREs group, and a 60 mg/mL RREs group were set in this study. After a culture of 48 hours, the medium was incubated with 10 mmol/L DCFH-DA at 37°C for 30 min and then washed with PBS for 3 times. After the treatment, pictures were taken under a fluorescence microscope immediately, and light should be avoided carefully during the operation.

### 2.7. Detection of Cell Mitochondrial Membrane Potential

The mitochondrial membrane potential of HepG2 cells in each group was detected by the method in reference [17]. After the cells were passaged, the cells were inoculated in a 96-well plate. When the cells were 60% full, they were processed. The drug treatment group was added with 30 mg/mL and 60 mg/mL RREs, and a serum-free medium was used for the control group. Six wells were repeated at each concentration and incubated for 48 hours in an incubator. The medium was discarded, and the wells were washed with sterile PBS for three times and added with 100 *μ*L JC-1 staining solution to stain for 30 minutes. After staining, sterile PBS was used to wash for 3 times, and 100 *μ*L of PBS was added and placed in a multifunction microplate detector to measure the fluorescence intensity. The decrease in the fluorescence intensity ratio indicated that the mitochondrial membrane potential decreased.

### 2.8. Western Blot

HepG2 cells were cultured routinely and inoculated in 6-well plates at 2 × 10^5^/well. After the liver cancer HepG2 cells were treated by RREs at different concentrations (30 and 60 mg/mL) for 48 hours, the cells in each group were collected and added with the cell lysis buffer to lyse the cells for protein extraction. The loading amount was 30 *μ*g. The protein was separated by 10% polyacrylamide gel electrophoresis and then transferred to the polyvinylidene fluoride (PVDF) membrane. The 5% skimmed milk solution was used to seal the plate at room temperature for 1 hour, and Bcl-2 (1 : 3000), Bax (1 : 3000) or Caspase-3 (1 : 3000), and *β*-actin primary antibody were added for incubation overnight at 4°C. After three times of washing with tris buffered saline tween (TBST) the next day, 1 : 2,000 diluted horseradish peroxidase-labeled rabbit secondary antibody was added and then incubated for 1 hour at room temperature. After the membrane was washed with TBST, the target band was detected by chemiluminescence method, and the band gray-scale analysis was performed. The experiment was repeated 3 times.

### 2.9. Statistical Analysis

SPSS 22.0 was employed for data processing, the data were expressed as mean ± standard deviation ($\bar{x}\pm s$), and the one-way analysis of variance was applied in this study. *P* < 0.05 indicated that the difference was statistically considerable.

## 3. Results

### 3.1. HepG2 Cell Activity

**Figure 1:**
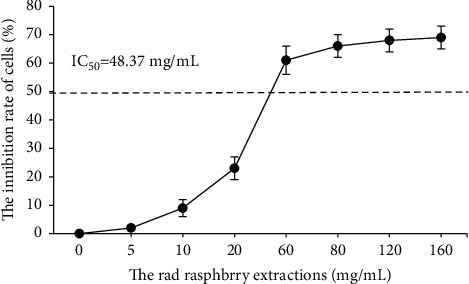
The inhibitory rate of different concentrations of RREs on the proliferation of liver cancer HepG2 cells.

After HepG2 cells were treated with different concentrations of RREs for 48 hours, RREs showed inhibitory effect on the activity of liver cancer HepG2 cells, which was concentration-dependent. The 50% inhibitory concentration of RREs against HepG2 cell activity was 48.37 mg/mL, while the lower concentration of RREs had no obvious inhibitory effect on cell proliferation (Figure 1).

### 3.2. HepG2 Cell Proliferation

**Figure 2:**
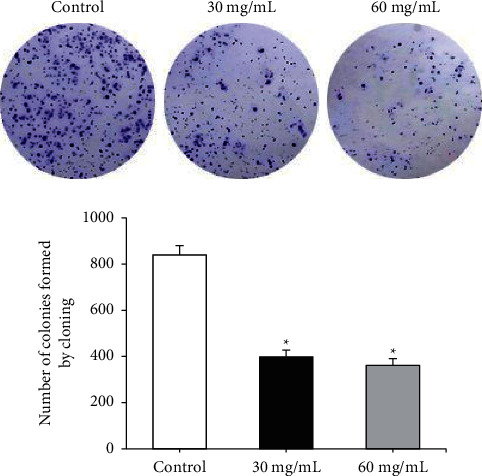
Effect of RREs on the proliferation of liver cancer cells determined by plate clone formation assay. ^*∗*^Compared to the control group, *P* < 0.05.

The results of the colony formation experiment revealed that the cells in the control group grew vigorously and the colonies were evenly dispersed. After being treated with RREs, the formation of the number of colonies was reduced steeply by RREs at a concentration of 30 mg/mL and 60 mg/mL (*P* < 0.05), and the effect of 60 mg/mL was more significant (Figure 2).

### 3.3. HepG2 Cell Apoptosis

**Figure 3:**
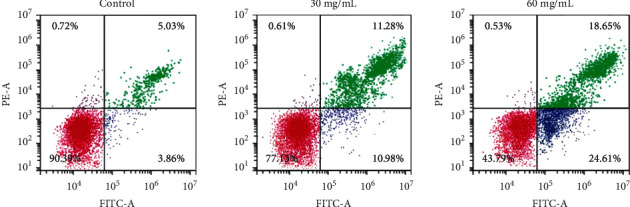
The effect of RREs on HepG2 cell apoptosis detected by flow cytometry.

Compared with the control group, both early and late apoptosis elevated obviously (*P* < 0.05) after the treatment of 30 mg/mL and 60 mg/mL RREs for 48 hours, which was concentration-dependent, suggesting that RREs treatment could induce HepG2 cell apoptosis (Figure 3).

### 3.4. ROS Measurement Results

**Figure 4:**
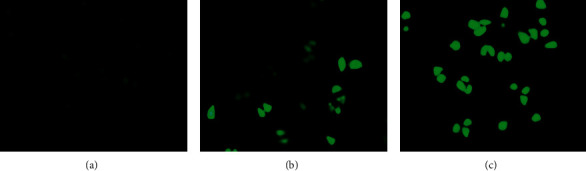
Effects of RREs on ROS production in HepG2 cells.

The change of intracellular ROS content is shown in Figure 4 after 30 mg/mL and 60 mg/mL RREs were used for treatment for 48 hours. Thus, RREs treatment could substantially increase the intracellular ROS level.

### 3.5. Measurement Results of Cell Mitochondrial Membrane Potential

**Figure 5:**
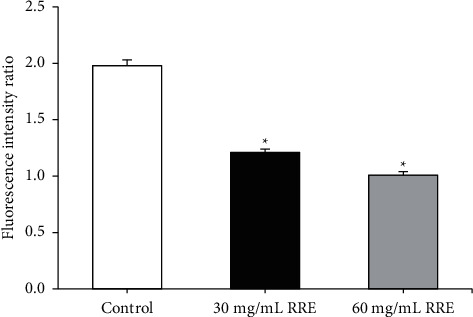
Effects of RREs on mitochondrial membrane potential reduction in HepG2 cells. ^*∗*^Compared to the control group, *P* < 0.05.

Figure 5 indicates that the RREs treatment for 48 hours decreased the mitochondrial membrane potential of HepG2 cells in a concentration-dependent manner after HepG2 cells were incubated with different concentrations of RREs.

### 3.6. Western Blot Test Results

**Figure 6:**
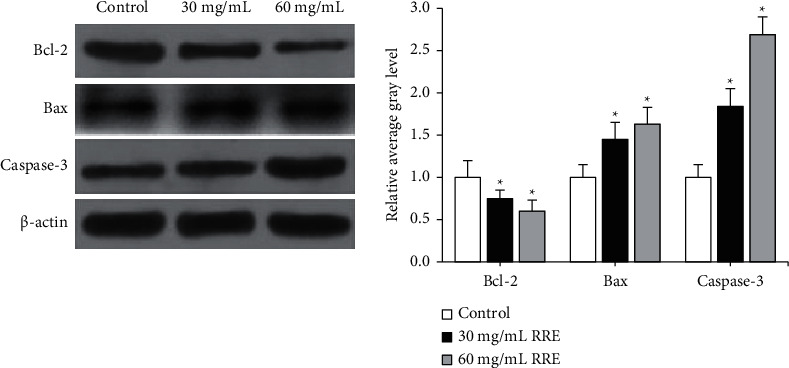
The effect of RREs on Bcl-2, Bax, and Caspase-3 protein expression detected by western blot. ^*∗*^Compared to the control group, *P* < 0.05.

To explore the specific mechanism of RREs promoting the apoptosis of liver cancer HepG2 cells, western blot was applied to detect the expression of Bcl-2, Bax, and Caspase-3. Compared with the control group, RREs could sharply inhibit the expression of Bcl-2 (*P* < 0.05) but enhance the expression of Bax and Caspase-3 (*P* < 0.05), thereby reducing the ratio of Bcl-2/Bax (Figure 6).

## 4. Discussion

Red raspberries are extensively applied in medical research and have certain effects on tumors, diabetes, and cardiovascular and cerebrovascular diseases [18]. Moreover, red raspberry juice or extractions have been proven to inhibit tumor growth in vivo and in vitro [19]. The experimental results of this study showed that RREs had a marked inhibitory effect on the proliferation of liver cancer cells in vitro. Besides, the inhibitory effect becomes more obvious, with the concentration growth of the RREs.

In recent years, research on red raspberry anticancer is more inclined to the specific molecular mechanisms of chemical composition anticancer, including research on signaling pathways, oxidative stress, cell cycle block, apoptosis, and angiogenesis [20, 21]. Lee et al. (2016) [22] pointed out that red raspberry component sanguiin H-6 (SH-6) could suppress the proliferation of ovarian cancer cells A2780. The experimental results suggested that SH-6 could pass P38, mitogen-activated protein kinase (MARK), and Caspase-8-dependent BID decomposition pathways to induce apoptosis of ovarian cancer cell A2780.

The proapoptotic and antiapoptotic proteins in the Bcl-2 family in normal cells are at a relatively stable level [23]. When cells are stimulated by apoptotic factors, the upstream apoptotic signaling pathway is activated, and proapoptotic proteins are activated and translocated to mitochondria [24]. The Bax gene induces mitochondrial fragmentation and the release of cytochrome C, which activates the Caspase cascade, leading to apoptosis [25]. What's more, Bcl-2 can inhibit the opening of mitochondrial permeability transition pore (MPTP), avoid the activation of the Caspase system, and prevent cell apoptosis [26]. The experimental results of this study indicated that RREs could reduce the expression of Bcl-2 protein in HepG2 cells, increase the expression of Bax protein to elevate the ratio of Bax/Bcl-2, promote the activation of Caspase-3, and cause cell apoptosis.

The key of cancer treatment is to inhibit the cancer cell proliferation and to induce cancer cell death, and ROS-mediated mitochondrial pathway apoptosis plays a critical role in the treatment of cancer [27]. Therefore, the ROS-mediated mitochondrial apoptosis pathway was used as a clue in this study to investigate the effect of RREs on mitochondrial membrane ROS production of liver cancer cells, and the effect of RREs induced ROS production on mitochondrial membrane potential of apoptotic cells, so as to reveal the effect and mechanism of RREs on liver cancer cells. From the research results, it could be inferred that RREs induced HepG2 cell apoptosis by generating ROS to disrupt the normal redox balance in the cell, which made the mitochondrial structure change, so as to disrupt the mitochondrial membrane potential and destroy the normal function of mitochondria, thereby leading to apoptosis of HepG2 cells. Studies have speculated that RREs may induce HepG2 cell apoptosis through ROS-mediated mitochondrial pathway.

## 5. Conclusions

To explore the therapeutic effect of RREs on hepatocellular carcinoma, HepG2 cells were treated with RREs, and the mechanism of its effect on cell proliferation and apoptosis was detected. The results suggested that RREs may inhibit the proliferation and apoptosis of HCC cells by inducing ROS production, decreasing Bcl-2/Bax ratio, and significantly decreasing mitochondrial membrane potential of HepG2 cells, thus inhibiting the progression of HCC. However, the in vitro assay was adopted to analyze the inhibitory effect of RREs treatment on HepG2 cells, without combining signal pathway or transcriptome data for specific analysis. In future studies, animal models of liver cancer will be further constructed and the specific effects of RREs on liver cancer will be verified in in vivo experiments. In conclusion, this study provides reference for finding new therapeutic methods for hepatocellular carcinoma.

## Data Availability

The data used to support the findings of this study are available from the corresponding author upon request.
